# Severe *Plasmodium vivax* malaria, HIV, tuberculosis co-infection in a Sri Lankan traveller: case management and challenges during the prevention of malaria reintroduction phase

**DOI:** 10.1186/s12936-018-2581-1

**Published:** 2018-11-16

**Authors:** Dewanee Ranaweera, R. M. J. Kanchana Rajapaksha, Priyanganie Silva, Raja Hettiarachchi, W. M. Kumudu T. de A. W. Gunasekera, Hemantha Herath, Deepika Fernando

**Affiliations:** 1 Anti Malaria Campaign, 555/5 Public Health Building, Narehenpita, Sri Lanka; 2 District General Hospital, Negambo, Sri Lanka; 3 Department of Parasitology, Faculty of Medicine, Kynsey Road, Colombo 8, Sri Lanka

**Keywords:** Severe, Plasmodium, Vivax, Malaria, Comorbid, Case management

## Abstract

**Background:**

The country received malaria-free certification from WHO in September 2016, becoming only the second country in the WHO South East Asia region to be declared malaria-free. Imported malaria cases continue to be reported, with 278 cases reported between 2013 and 2017. The diagnosis of a severe *Plasmodium vivax* patient co-infected with HIV and tuberculosis is discussed with an overview of the rapid response mounted by the Anti Malaria Campaign (AMC), Sri Lanka.

**Case presentation:**

A Sri Lankan gem miner who returned from Madagascar on the 6th of April 2018 presented to a private hospital for a malaria diagnostic test on the 21st April, 2 days after the onset of fever. He came on his own for this test due to the awareness he had regarding the risk of imported malaria. As the patient was positive for *P. vivax* malaria, he was admitted to a government hospital for further management. The patient had features of severe malaria upon admission with a systolic BP < 80 mmHg and thrombocytopaenia (38,000 cells/mm^3^). Treatment with IV artesunate was initiated immediately and management was carried out rapidly and efficiently by the clinicians with guidance from the staff of the AMC headquarters, which resulted in a rapid recovery of the patient. IV artesunate was followed by a course of artemether plus lumefantrine and the blood smear was negative for malaria by the 2nd day. A 14-day course of primaquine was commenced after excluding a G6PD deficiency. Due to an accidental needle stick injury of a health care worker attending on the patient was tested for HIV and subsequently tuberculosis and was found to be positive for both infections. The patient was discharged on the 1st of May with instructions for follow up visits for malaria. Management of the HIV and tuberculosis infections was attended to by the clinicians and staff of the appropriate disease control programmes (i.e. the national STD/AIDS Control Programme in Sri Lanka and the National Programme for tuberculosis control and chest diseases).

**Conclusions:**

It is important to consider comorbid conditions and immunosuppression when a patient with a benign form of malaria presents with severe manifestations. Measures should be strengthened to prevent importation of diseases, such as malaria and AIDS through migrant workers who return from high-risk countries.

## Background

Sri Lanka was certified as a malaria-free country by World Health Organization in 2016. Prevention of re-introduction of malaria and maintaining zero mortality due to malaria are current objectives of Anti-Malaria Campaign (AMC), Sri Lanka. Early diagnosis and proper management of malaria infections is a key strategy in achieving these objectives. Since the last indigenous malaria case was diagnosed in Sri Lanka in October 2012, only imported malaria cases have been reported in Sri Lanka, totalling 278 cases from 2013 to 2017. In spite of severe malaria patients being reported in the country (e.g. three severe malaria cases in 2017, all due to *Plasmodium falciparum*), zero mortality has been maintained since 2007.

Severe malaria is most commonly caused by *P. falciparum* infections, but *Plasmodium vivax* [1–6] and *Plasmodium knowlesi* [7, 8] has also been increasingly reported as causing severe disease. The risk of severe *P. vivax* malaria is high among children and people with comorbid conditions [9]. This publication describes a severe *P. vivax* case in Sri Lanka in a patient with HIV and tuberculosis, illustrating the rapid response mounted in response to it, and discusses case management and challenges faced in a country that has eliminated malaria.

## Case presentation

**Fig. 1:**
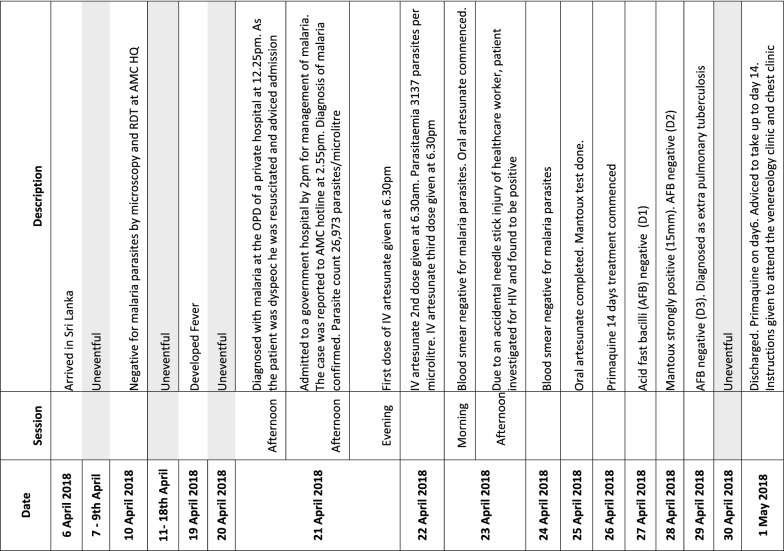
Time line of important clinical events

A 36 year-old Sri Lankan national and gem miner by occupation who returned to Sri Lanka from Madagascar on the 6th of April 2018, visited the AMC headquarters 4 days after his arrival for a malaria diagnostic test (Fig. 1). Even though both microscopy and the rapid diagnostic test (RDT) were negative for malaria, the attending medical officer at AMC headquarters advised him to get tested for malaria if he developed fever subsequently. The patient developed fever on the 19th of April. Remembering the advice given at AMC headquarters, he requested 2 days later a blood smear for malaria diagnosis at a private health care institute situated in his home town, a previously non-endemic area for malaria of the Western Province. The blood smear reported positive for malaria parasites (*P. vivax*).

As the patient had dyspnoea at the time of presentation he was resuscitated with oxygen and requested to get admitted. The patient got admitted immediately to a government hospital in the same district for management of the malaria infection. He complained of severe headache and fatigue at admission (D0). On examination the patient was afebrile (98.4 °C), had a heart rate of 116 (tachycardic), blood pressure of 80/54 mmHg (which 4 h later reduced to 74/44 mmHg) and had dyspnoea (with a respiratory rate of 28, O_2_ saturation was normal). He was centrally cyanosed but conscious and rational with a Glasgow coma scale at 15/15. There was no pallor, jaundice or hepatosplenomegaly. Figure 1 gives the time lines in relation to the illness of this patient.

**Fig. 2:**
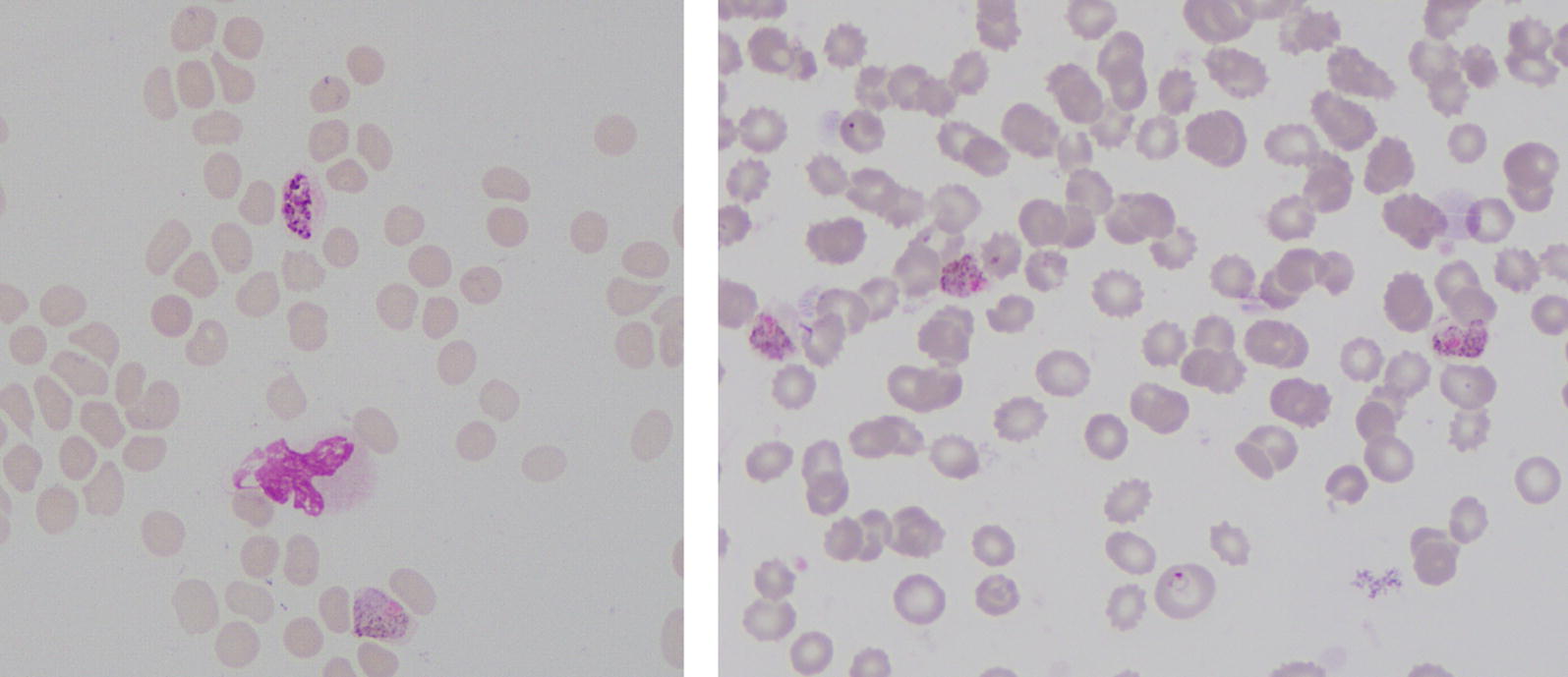
Blood smear showing trophozoite stages of *P. vivax*

The case was reported to the Anti Malaria Campaign (AMC) telephone hotline at 2.55 pm. In spite of being a weekend, the staff members’ on-call was rapidly mobilized to attend to the patient. The diagnosis of *P. vivax* malaria was confirmed by examination of thick and thin Giemsa-stained blood smears by the Public Health Laboratory Technician (PHLT) of the government hospital. At admission (day 0) parasitaemia of 26,973 parasites per microlitre of blood was reported with the presence of rings, trophozoites and gametocytes. The morphology of the infected red blood cells was compatible with a *P. vivax* infection (Fig. 2). A RDT performed by the PHLT also gave a positive pLDH band. Results of PCR assay which was done at the Central laboratory of the AMC confirmed a mono-infection with *P. vivax*.

Laboratory investigations showed normal haemoglobin concentration (13.4 g/dl, reference range 11–16.5), which subsequently reduced to 9 g/dl by day 6 of admission), with a slightly elevated leukocyte count 12,120 (reference range 4000–11,000) (N 83%, L 14%), severe thrombocytopaenia (38,000 cells/mm^3^, reference range 150,000–400,000 cells/mm^3^) and a normal capillary blood sugar level of 124 mg/dl (reference range 100–200 mg/dl). There were features of acute renal failure as the serum creatinine levels increased steadily from the time of admission 1.17 mg/dl (at admission) to 1.37 mg/dl (at 2 pm the same day) to 2.27 mg/dl (at 6 pm the same day) (reference range 0.8–1.2 mg/dl) with a reduced urine output. The C Reactive protein level was elevated (73. 8 mg/l, reference range 0–3 mg/l). His liver enzymes levels were SGPT 53U/L (10-40), SGOT 59 U/l (13-31), bilirubin level 5.03 mg/dl (0.1–1.2 mg/dl). Ultra sound scan abdomen showed a mild splenomegaly (BPL 12 cm) and no hepatomegaly was detected. Blood culture was negative for microorganisms.

Following good hydration with IV normal saline and IV noradrenaline infusion for 24 h, his blood pressure stabilized and serum creatinine returned to the normal level (1.22 mg/dl) with improved urine output by the next day. For the management of malaria, the AMC medical officer on-call visited the patient with IV artesunate (2.4 mg/kg body weight) and treatment was initiated by the ward medical staff based on the national treatment guidelines under the guidance of AMC consultant, with the first dose being administered within 4.5 h of admission to the hospital and two more doses given 12 hourly apart the next day. Following this, oral artemisinin-based combination therapy (artemether–lumefantrine) was commenced and continued for 3 days. With the commencement of treatment, the parasitaemia reduced to 3137 parasites per microlitre the next day (day 1) and the blood smear was negative for parasites by day 2. A 14-day course of primaquine was initiated on the sixth day after excluding a G6PD deficiency.

Following a needle prick injury to an attending health care worker, consistent with a circular issued by the Ministry of Health, Sri Lanka, HIV screening was carried out in this patient and he was found to be positive [10]. HIV status was confirmed by ELISA which was also positive. Further confirmation of the HIV status was done by Western blot technique. Screening for tuberculosis indicated that sputum was negative for acid-fast bacilli, but the Mantoux test was strongly positive (15 cm). Due to the presence of a bluish discolouration of his nails, mild dyspnoea, persistent tachycardia with recurrent fever spikes since the 3rd day of admission (with repeat blood smears for malaria parasites being negative) and a rising CRP (130 mg/dl), a *Pneumocystis carinii* infection was suspected. Even though the chest X-ray was normal, the patient was started on oral cotrimoxazole (4 tablets stat and 3 tablets three times a day for 21 days). IV caftazidime was also started along with oral cotrimoxazole to cover other possible infections.

**Fig. 3:**
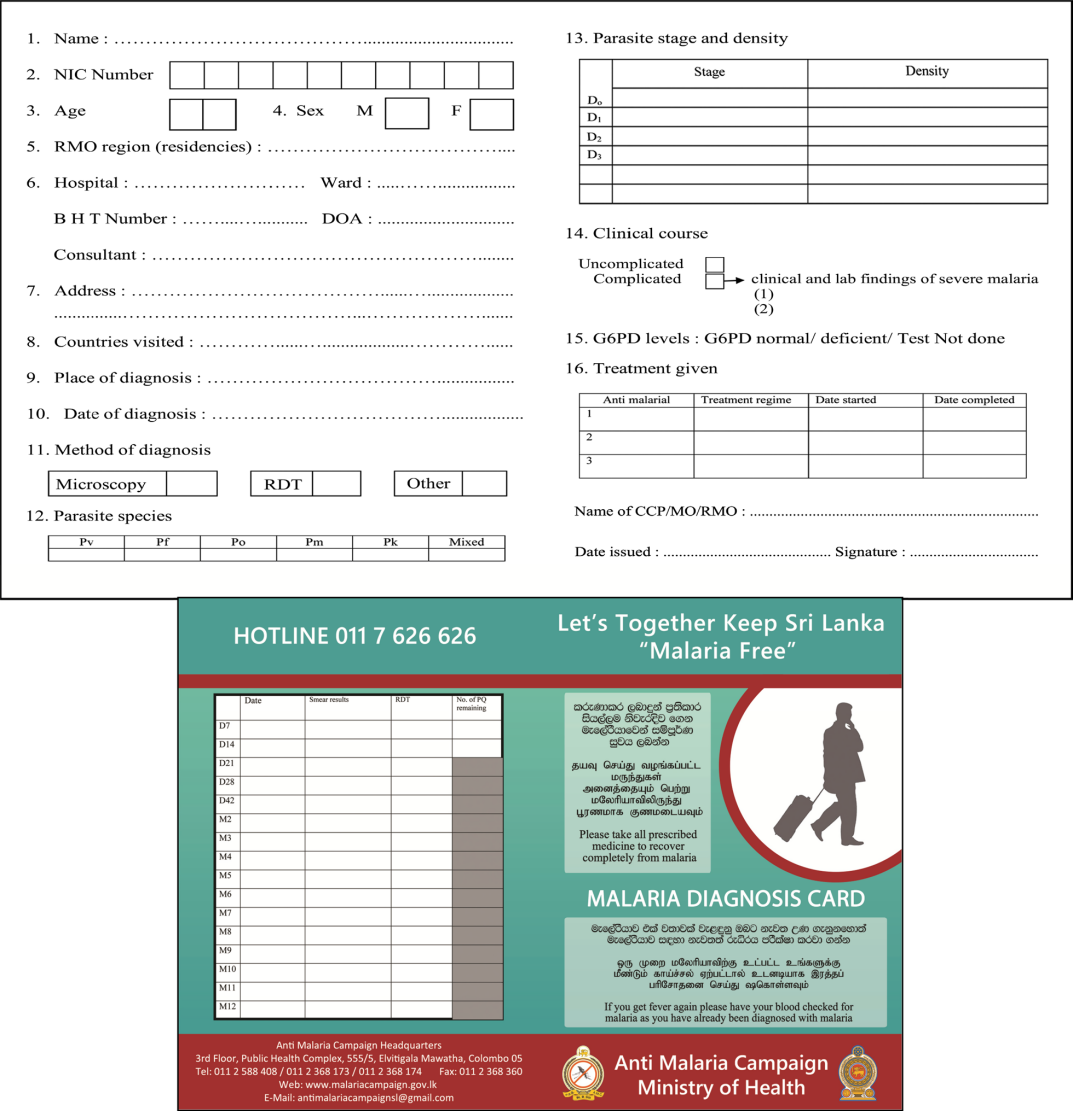
.

The patient was discharged from the hospital on the 1st of May with a malaria diagnosis card issued by the AMC. The especially designed card highlights the importance of completing the treatment and the indicate follow up dates to confirm the absence of malaria parasites (Fig. 3). Both the Public Health Inspector (PHI) of the patients’ area of residence and the PHI from AMC headquarters ensured that the balance primaquine was taken as required by home visits. Management of the HIV and tuberculosis infections was dealt with by the attending physicians in collaboration with the appropriate disease control programmes (i.e. the national STD/AIDS Control Programme in Sri Lanka and the National Programme for tuberculosis control and chest diseases).

The patient had left Sri Lanka for Madagascar on the 26th of December 2017, but had not taken chemoprophylaxis, but indicated that he slept under a mosquito net. The patient who is a frequent visitor to Madagascar since the year 2005, initially obtained malaria prophylaxis, but stopped taking the drugs as he would have to use the prophylactic medicines long term. He gave a past history of several episodes of malaria with the last episode being in August 2017.

## Malaria case investigation and response

Case investigation which comprises both parasitological and entomological surveillance to ensure that no onward malaria transmission takes place commenced on the same day. A detailed history obtained from the patient indicated the presence of two other contacts who had returned from Madagascar with the patient. On contact tracing (a form of reactive case detection practiced in Sri Lanka), one individual had already been diagnosed with malaria in December 2017 and was being followed up by the AMC. The other person, who returned with the above patient was negative for malaria at the time. Reactive case detection by parasitological screening was carried out in 154 neighbourhood contacts of the patient according to standard surveillance guidelines and procedure of the AMC.

Entomological surveillance was conducted around the residence of the patient, but *Anopheles culicifacies*, the primary vector of malaria in the country was not found. However, three (0.6 per 100 dips) larvae and one adult of *Anopheles subpictus,* the secondary vector were detected. Larvivorous fish were introduced to the cemented pond where *An. subpictus* larvae was detected and a round of night fogging with insecticides was conducted to destroy any adult mosquitoes.

Public and health care workers in the vicinity were made aware of the need to be vigilant on malaria.

## Discussion

This is the first case of severe *P. vivax* malaria with HIV and tuberculosis co-infection reported from Sri Lanka. Maintaining zero mortality due to malaria in Sri Lanka since 2007 is a noteworthy achievement considering that in most countries where imported malaria cases are diagnosed, fatalities due to imported malaria are reported. In China, between 2011 and 2015, 8653 imported *P. falciparum* cases were diagnosed leading to 98 deaths (11.3 per 1000 cases) [11], while in UK, of 1400 cases reported in 2015, and 1618 in 2016, six deaths were reported each year [12]. Stepien and Rosinska [13] have reported of fatalities due to imported falciparum malaria in Poland due to delay in seeking medical care and delay in diagnosis. Operation of rapid response teams, maintaining a 24-h telephone hotline, strategic distribution of antimalarial drugs to reach a patient within a few hours of diagnosis of malaria, guidance provided to the attending physicians on case management and creating awareness among health care workers and high risk groups are some of the key activities implemented by the AMC, Sri Lanka to ensure early detection and proper case management of malaria which in turn contributes to maintaining zero mortality.

**Fig. 4:**
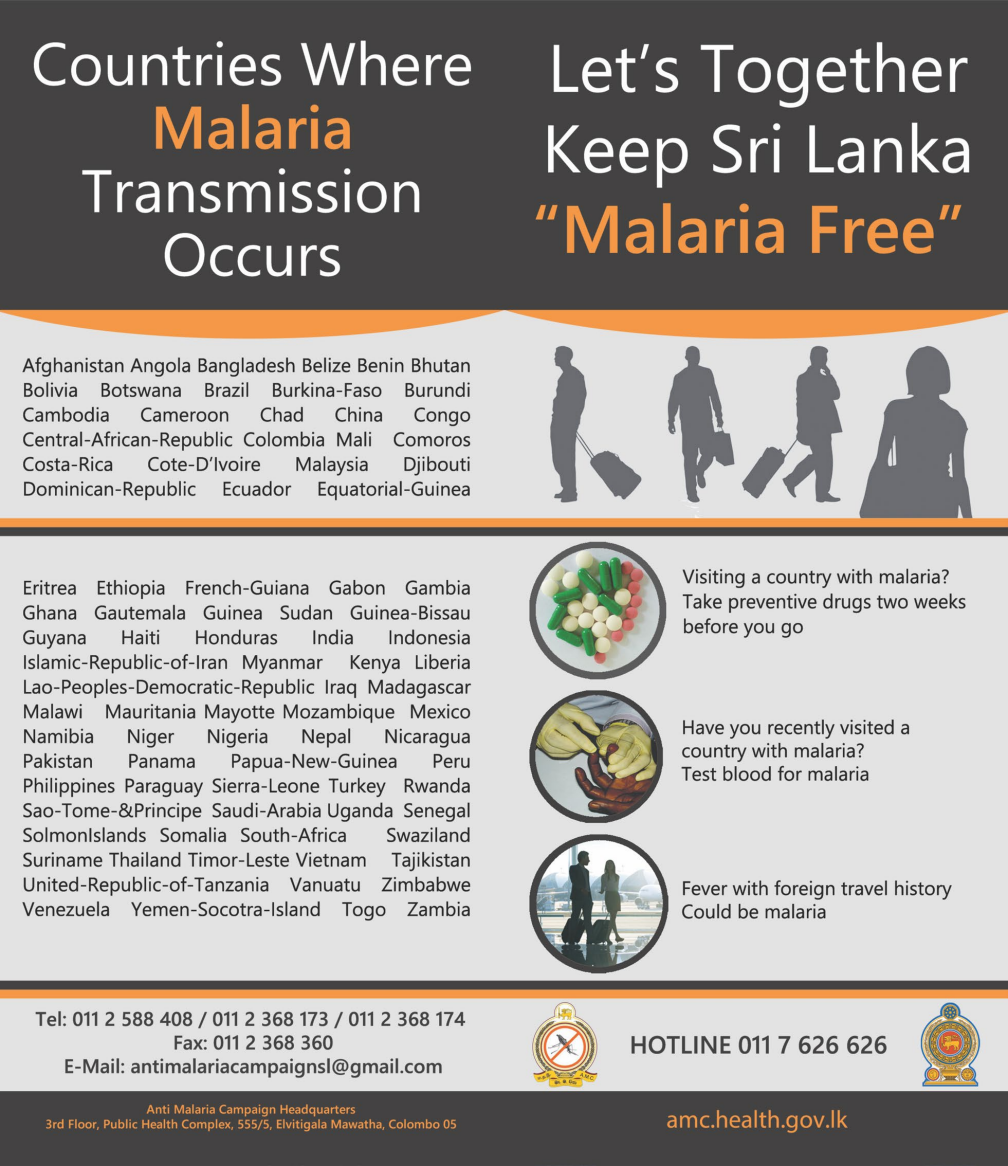
A ticket holder issued to individuals travelling to malaria endemic countries in an attempt to increase awareness regarding imported malaria. Designed and made available by the Anti-Malaria Campaign, Sri Lanka

Regular active case detection along with intensive awareness programmes carried out by the AMC amongst high-risk groups such as gem miners employed in African countries has made travellers aware of the need to get a blood smear examined for malaria upon arrival in Sri Lanka or if they develop fever within a year of their return. In 2017 alone, nine imported malaria cases were reported amongst Sri Lankans arriving from Madagascar. Awareness regarding mechanisms to prevent imported malaria has been further strengthened by providing informative printed material, such as airline ticket holders prior to departure (Fig. 4), and visually displaying key messages at the arrival lounge at the Bandaranaike International Airport. It is compulsory for the travellers to African countries to get vaccinated against yellow fever before their travel which is provided at the Port Health Office in Sri Lanka. The AMC works in close collaboration with staff of the Port Health Office to ensure that travellers visiting to obtain the yellow fever vaccination are referred to AMC. The AMC will then provide education, advice and guidance on the risk of malaria and preventive measures including the provision of medicines for chemoprophylaxis free of charge. These mechanisms led to this patient seeking the assistance of the AMC headquarters to get himself screened for malaria immediately after arrival on 6th of April, and again to seek a malaria diagnosis on developing fever 13 days later. This patient had not obtained chemoprophylaxis for malaria prior to departure. High-risk groups not only stay for a long duration of time but also travel between the two countries frequently. Providing prophylaxis to these long-term travellers is another challenge faced by AMC since adherence and tolerability are important aspects to be considered in long-term chemoprophylaxis. Only few studies have been reported on the use of chemoprophylaxis for more than 6 months [14].

In spite of a negative blood smear in the first instance, which could have been due to the presence of only liver stage of *P. vivax* at that point or undetectable levels of blood parasitaemia, the patient was vigilant enough to once again seek a diagnosis of malaria at a private health care institute when he developed fever, where malaria was promptly diagnosed. This reflects the impact of frequent training programmes carried out by AMC for the private health sector on malaria diagnosis. In the absence of evidence of locally-transmitted malaria in Sri Lanka and the patient having been in Madagascar recently it is very likely that the patient acquired malaria infection in Madagascar. Not only is *P. vivax* endemic in Madagascar [15], but also UNAIDS estimates that the number of people infected with HIV is rising steadily in that country [16].

The AMC mounted a rapid response in confirming the diagnosing and managing this patient. On receiving information on the 24-h telephone hotline of the AMC that a malaria positive patient was detected 40 km away from the AMC headquarters, the medical officer on call visited the patient, ensured that the diagnosis was confirmed and provided antimalarial medicines. The AMC is the sole importer and stockiest of anti-malarial medicines in the country. Due to the low incidence of imported malaria in the country and hence the very low usage of anti-malarial medicines, stocks are maintained only at the AMC and major government hospitals. For other health care institutions anti-malarial medicines are delivered to the clinicians immediately on request. This also ensures that any suspected malaria case is immediately reported to AMC headquarters whose consultants provide guidance on case management on all reported malaria cases based on the National Malaria Treatment guidelines [17]. Following treatment the patient made a full recovery of his malaria infection. Health care personnel serving around the area of residence of the patient were informed of the presence of a malaria positive patient and the need to rule out malaria in patients presenting with fever. To ensure early detection and proper case management by clinicians in the face of a very low number of malaria cases in the country, regular clinical awareness programs are carried out by the AMC and information is provided through the newsletter of the Sri Lanka Medical Association [18, 19].

This malaria infection was classified as severe based on the fact the patient had a systolic BP < 80 mmHg, acute kidney failure [20] and thrombocytopaenia (platelet count < 50,000 cells/mm^3^). Severe thrombocytopaenia (< 50,000 cells/mm^3^) has been reported as the most common “severe” manifestation among 77 studies reporting severe vivax malaria [21]. In the presence of this low platelet count (38,000 cells/mm^3^), should this patient not have requested a malaria diagnostic test at the private laboratory, it is very likely that dengue infection may have been the first differential diagnosis of the clinicians, and that malaria would have been overlooked, as has happened in many cases of imported malaria in the country in the past few years. This is because of an on-going dengue epidemic in the country with over 186,101 cases and 413 deaths reported in 2017 [22]. The severity of the malaria infection would have been very likely due to the concurrent HIV infection and the resulting immunocompromised state in the patient. The HIV co-infection in this patient was detected during a routine investigation for a needle stick injury to the attending health worker.

Sri Lanka has been able to prevent the re-introduction of indigenous malaria for over 5 years since October 2012 due to the many strategies it implements—awareness programs, a rapid response on detection of a malaria case, and intensive case investigation and management of patients by the AMC in addition to entomological surveillance and response. This is a noteworthy achievement given the challenges of high receptivity in a tropical country and the increased vulnerability afforded by increased foreign travel among Sri Lankans to malaria endemic countries and tourists and visitors, including foreign labour, from malaria endemic countries. The situation calls for continued vigilance to prevent the re-introduction of malaria to the country.

## Conclusions

This manuscript highlights the importance of mechanisms in place to sustain malaria elimination in Sri Lanka and the key strategies followed by the Anti Malaria Campaign of Sri Lanka when a malaria case is reported. It is important to consider comorbid conditions and immunosuppression when a patient with benign forms of malaria present with severe manifestations. Measures should be strengthened to prevent importation of diseases such as malaria and AIDS through migrant workers who travel to high-risk countries.

## Abbreviations

AMC: Anti Malaria Campaign
pLDH: parasite lactate dehydrogenase
PCR: polymerase chain reaction
PHI: Public Health Inspector
PHLT: Public Health Laboratory Technician
RDT: rapid diagnostic test
WHO: World Health Organization
